# Atomoxetine Treatment of Attention Deficit/Hyperactivity Disorder Symptoms in 3–6-Year-Old Children with Autism Spectrum Disorder: A Retrospective Cohort Study

**DOI:** 10.3390/children11020163

**Published:** 2024-01-26

**Authors:** Hamza A. Alsayouf, Osama Alsarhan, Wael Khreisat, Azhar Daoud

**Affiliations:** 1 Dr Hamza Alsayouf Clinic, Amman 11181, Jordan; dr.osama91alsarhan@gmail.com; 2 Royal Medical Services, Amman 11855, Jordan; wael_khreisat@yahoo.com; 3 The Specialty Hospital, Amman 11942, Jordan; ped-program-dir@specialty-hospital.com

**Keywords:** atomoxetine, attention deficit/hyperactivity disorder, autism spectrum disorder, adverse events, safety

## Abstract

Atomoxetine is indicated for the management of attention deficit/hyperactivity disorder (ADHD) in children and adolescents aged 6 to 18 years. Few studies have assessed the safety and tolerability of atomoxetine in younger patients. This retrospective cohort study included 133 children aged 3–6 years who were diagnosed with ADHD comorbid with autism spectrum disorder (ASD). The primary endpoint was the evaluation of the safety profile of atomoxetine. In total, 50 patients (37.6%) experienced adverse events (AEs), which led to treatment discontinuation in 23 patients (17.3%). The most common AEs were gastrointestinal (24.1%), aggression or hostility (12.8%), and increased hyperactivity (9.0%). In the 23 patients who discontinued treatment, all the AEs resolved after treatment ceased. Among the 110 patients who completed at least 6 months’ treatment, atomoxetine titrated to a dose of 1.2–1.8 mg/kg/day appeared to be well tolerated and effective. The Clinical Global Impression—Improvement score improved to 1 (“very much improved”) and 2 (“much improved”) in 62.4% and 20.3% of children, respectively, at their last visit. Overall, atomoxetine appeared to be well tolerated in younger children with comorbid ADHD and ASD. Nevertheless, close patient monitoring remains essential, and the study limitations necessitate caution in generalizing these findings to broader populations. Long-term prospective studies are required.

## 1. Introduction

Attention deficit/hyperactivity disorder (ADHD) is a common comorbidity in children with autism spectrum disorder (ASD) [1] and the most commonly diagnosed psychiatric condition in childhood and adolescence [2]. It is characterized by inattention, hyperactivity, and impulsivity [3], which negatively affect several life domains, including socialization and educational outcomes; these effects extend into adulthood, having lasting impacts on quality of life [4]. The prevalence of ASD is increasing [5], and, given that ADHD comorbidity occurs in 40–70% of these patients [1], there is a growing need for safe and effective treatment strategies to help improve young patients’ neurodevelopmental outcomes.

There are similarities in symptoms between ADHD and ASD, and children with comorbid ADHD/ASD tend to be diagnosed with ADHD first and may experience a delay in ASD diagnosis compared with those without comorbid ADHD [6]. This overlap in symptoms may be underpinned by similarities in neuropathology, as these conditions share alterations in, for example, sensory processing, androgen metabolism, motor and impulse control, neural connectivity, and sleep patterns, which suggest a related etiology [7,8,9].

Updates to the 5th edition of the Diagnostic and Statistical Manual of Mental Disorders (DSM-5) criteria now allow the diagnosis of children with milder ADHD symptoms and comorbid diagnosis with other conditions such as ASD [3,10]. This should enable earlier and broader diagnosis of comorbid ADHD/ASD and inform more appropriate treatment strategies for affected children [2,10,11]. Importantly, the standard rating scales used to assess ADHD have not yet been validated for patients with comorbid ASD, although these scales remain useful in monitoring treatment outcomes [11]. Thus, the physician needs to carefully consider differential diagnoses of ADHD in the context of ASD, given that children with ASD may appear to display inattention and hyperactivity that are attributable instead to the delayed language development and perseverative behaviors typical of ASD [11]. This requires close observation of the child in a variety of settings and obtaining reports from not only parents/guardians but also teachers, caregivers, and other clinicians involved in the child’s care. Compared with their unaffected peers, children with ADHD tend to have difficulties in developing fine motor abilities (such as writing), basic mathematics skills, and prereading skills [4,12]. These children are also faced with socialization and behavioral challenges, such as aggression, disruptiveness, and impulsivity, which can raise safety concerns for themselves and their peers and lead to expulsion from preschool/daycare settings. In children with comorbid ASD, these issues can exacerbate and perpetuate impairments and place further considerable strain on families and caregivers [11]. Furthermore, early-onset ADHD is known to be a strong risk factor for later delinquency, criminality, mental illness, substance abuse, and poor school and job performance [13].

It remains to be elucidated whether the underlying pathology of ADHD can be ameliorated through early intervention, for example by taking advantage of the higher neuroplasticity of the brain in early childhood [13,14,15,16]. However, there are other potential benefits in addressing ADHD symptoms as soon as possible [13]. These include preventing the development of negative behaviors and habits that may later worsen impairments, preventing the hardening of parent/caregiver attitudes towards the management of ADHD, and preempting difficulties at school and the negative cycle of low self-esteem and poor performance that may result.

ADHD has been linked to dysfunctional neuronal activity in several areas of the brain, particularly those involved in attention and executive functions [17]. Non-pharmacological treatment strategies, such as parent training and behavioral classroom interventions, can be of benefit in younger children; however, these strategies are not always sufficiently effective in the management of ADHD symptoms, and the presence of comorbidities such as ASD can further complicate treatment [2]. Pharmacological therapies that are used to treat ADHD and may also have a beneficial effect in patients with comorbid ASD include stimulants such as methylphenidate, selective norepinephrine reuptake inhibitors such as atomoxetine, α-2 adrenergic agonists such as guanfacine and clonidine, and antipsychotics such as risperidone and aripiprazole [18].

Atomoxetine has been shown to be effective in the treatment of ADHD in both children and adults [19], and its mechanism of action is thought to be the specific presynaptic inhibition of norepinephrine (NA) reuptake by NA transporters [20]. Atomoxetine is currently approved for the treatment of ADHD in children aged 6 years and older [21]. Despite evidence indicating the early onset of ADHD symptoms in children as young as 3 years of age and the need for interventions in preschool children [2,11,13,19], few studies have assessed the use of atomoxetine in children younger than 6 years old.

In this retrospective cohort study, we report our clinical experience using atomoxetine in children of 3–6 years of age with comorbid ADHD and ASD. The primary endpoint was to evaluate the safety profile of atomoxetine in this cohort, and the secondary endpoint was to assess the drug’s effectiveness in managing the symptoms of ADHD. Among the 133 patients included in this study, 23 patients discontinued treatment due to adverse events, all of which resolved after treatment ceased. In the 110 patients who completed at least 6 months’ treatment, atomoxetine titrated to a dose of 1.2–1.8 mg/kg/day was found to be well tolerated and effective.

## 2. Materials and Methods

### 2.1. Study Design and Setting

This retrospective cohort study was performed in children aged 3–6 years with a diagnosis of ADHD associated with ASD. Participants in the study were retrospectively recruited based on chart review and were selected from families who visited the child neurology clinics of the authors in Jordan between 2021 and 2023. All patients selected for this study had not responded previously to supportive therapies or did not have access to these, and pharmacological intervention was recommended (supportive therapies comprised behavioral therapy for children and/or parent training on behavior management from a licensed therapist where accessible).

### 2.2. Participants

Eligibility criteria for inclusion were as follows: (1) met the DSM-5 criteria for ADHD in conjunction with ASD [3]; (2) diagnosis was established by a child neurologist and clinical psychologist; (3) score of ≥4 on the Clinical Global Impression—Severity (CGI-S) scale for ADHD [22]; (4) no known history of other chronic medical illnesses or conditions (including gross motor delay, intellectual disability, or cerebral palsy); (5) not taking any medications or supplements other than atomoxetine; (6) parents/legal guardians had provided informed consent prior to starting treatment regarding the potential anonymous use of their child’s data in a retrospective chart review; and (7) minimum duration of 6 months’ atomoxetine treatment.

The diagnostic process was based on data collected through direct observations of the patients’ behaviors, historical data, and the use of the DSM-5 criteria along with supportive standardized scales that included the Childhood Autism Rating Scale—2nd Edition Standard form (CARS2-ST) [23] and the Vanderbilt ADHD diagnostic parent rating scale [24] for the diagnosis of ASD and ADHD, respectively. All parents/legal guardians were counseled regarding the potential adverse effects of atomoxetine and the off-label use of this medication in this age group.

At baseline, an auditory brainstem response (ABR) test, electrocardiogram (ECG), and electroencephalogram (EEG) were performed for each patient as part of the ASD evaluation process to exclude other potentially treatable conditions requiring alternative treatment strategies [25,26,27]. The ABR test was used to assess any hearing loss, and an EEG with a sleep sample was performed to evaluate for electrical status epilepticus in sleep or epileptic encephalopathy. In addition, an ECG was obtained before starting atomoxetine to check the corrected QT interval (QTc) [2,28].

### 2.3. Ethics Approval

This study was approved by the independent Pearl Institutional Review Board (IRB) as an exemption and followed the ethical standards of the 2000 revision of the 1975 Declaration of Helsinki. The IRB approval number is 20-KNRC-101.

### 2.4. Treatment

Atomoxetine doses, starting at 0.5 mg/kg, were slowly increased by 0.5 mg/kg every 2–4 weeks, as tolerated, up to a maximum dose of 1.8 mg/kg/day. This dosing regimen was informed by the atomoxetine package insert [21] and findings from previous studies of atomoxetine in children younger than 6 years of age [29,30,31]. Since an oral atomoxetine solution is not available in Jordan, parents were directed to dissolve the powder of atomoxetine capsules (dose [mg] dependent on child’s weight) in 5 mL water to facilitate administration in these younger patients. Patients received this solution of atomoxetine once daily in the mornings. Parents were directed to administer the first dose of atomoxetine and implement any dose increments over the weekend to allow parents to monitor for any potential adverse events and side effects.

### 2.5. Study Outcomes and Analysis

The primary endpoint was to assess the safety profile of atomoxetine based on: (1) an adverse events checklist completed by parents/caregivers (this included a list of the common adverse events reported for atomoxetine based on the atomoxetine package insert [21]); (2) a side effects review (data collected from staff interviews with parents/caregivers about possible side effects); (3) clinical observations; (4) vital signs (blood pressure, pulse, height, weight); and (5) laboratory assessments (complete blood count and kidney and liver function tests). The adverse events checklist, side effects review, and vital signs measurements were performed at each clinic visit. The laboratory tests were performed at baseline and every 6 months. After starting medications, any new behaviors known to be a side effect of atomoxetine treatment, or any significant worsening of existing symptoms from baseline, were considered related to atomoxetine treatment.

The secondary endpoint of the study was an evaluation of the effectiveness of atomoxetine in the treatment of ADHD comorbid with ASD in this pediatric cohort. Any changes (improvements or worsening) in the patients’ ADHD symptoms were assessed using the Clinical Global Impression—Improvement (CGI-I) scale [22] based on interviews with families and caregivers and verified by clinical observations during follow-up visits.

All analyses of outcomes data were descriptive only (frequency distributions and percentages), and no statistical testing was performed.

## 3. Results

### 3.1. Patient Disposition, Baseline Demographics, and Clinical Characteristics

Between 2021 and 2023, a total of 133 children met the eligibility criteria for this retrospective cohort study (full analysis set). At baseline, all children were aged 3 to 6 years old (mean, 5 years 4 months), with a weight of 12.5 to 47.2 kg (mean, 21.8 kg). The patients were mostly male (*n* = 114; 85.7%) and Arabic (*n* = 95; 71.4%); 11 were Indian (8.3%), 7 were Asian (5.3%), 4 were North American (3.0%), 3 were Iranian (2.3%), 2 were European (1.5%), and 11 were of other races/descents (8.3%). All participants were diagnosed with ASD with comorbid ADHD. The types of ADHD present were primarily inattentive (*n* = 39, 29.3%), primarily hyperactive (*n* = 13, 9.8%), and combined (*n* = 81, 60.9%). The duration of treatment ranged from 6 months up to 25 months, with a mean of 17 months.

### 3.2. Safety Outcomes

**Figure 1:**
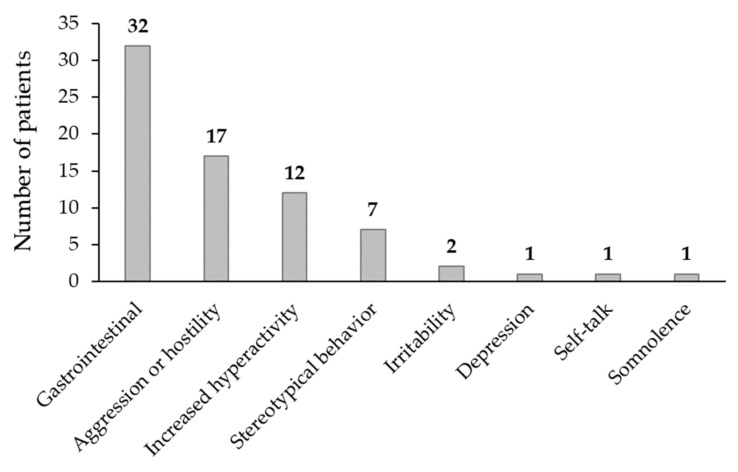
Adverse events and side effects reported in the 133 patients who received ≥6 months of atomoxetine treatment.

During the course of treatment, a total of 50 of 133 patients (37.6%) reported various adverse events and side effects, including the worsening of symptoms (Figure 1). These events were sometimes noted to be dose dependent. Gastrointestinal adverse events (appetite loss, nausea, vomiting, and abdominal pain [*n* = 32; 24.1%]), aggression or hostility (*n* = 17; 12.8%), and increased hyperactivity (*n* = 12; 9.0%) were the most commonly reported.

In 23 patients (17.3%), adverse events were deemed sufficiently severe to recommend discontinuation of atomoxetine treatment. The most common adverse events leading to discontinuation were aggression/hostility (*n* = 13) and increased hyperactivity (*n* = 12); these adverse events were reversible and resolved following cessation of treatment. All other adverse events and side effects were milder in severity and were managed conservatively by reducing dose titration from every 2 weeks to every 4 weeks.

### 3.3. Effectiveness

**Figure 2:**
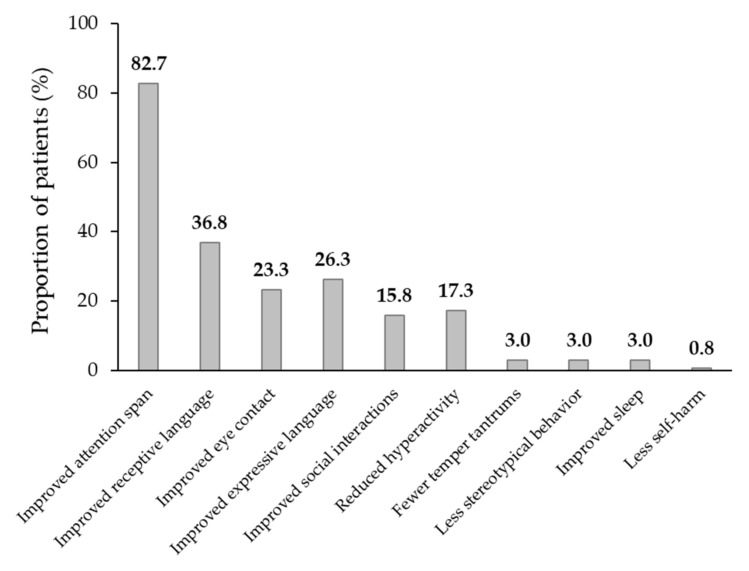
Effect of ≥6 months of atomoxetine treatment on attention deficit/hyperactivity disorder symptoms based on the Clinical Global Impression—Improvement (CGI-I) scale (*n* = 133).

All 110 of the 133 patients (82.7%) who completed at least 6 months of atomoxetine treatment displayed improvements in their ADHD and/or ASD symptoms based on the CGI-I scale (Figure 2). All 110 (82.7%) had improvements in their attention span, 49 (36.8%) had improvements in their receptive language and the ability to follow commands, 31 (23.3%) had improved eye contact, 35 (26.3%) had improvements in their expressive language, 21 (15.8%) had improved social interactions, 23 (17.3%) had reductions in hyperactivity, 4 (3.0%) had reductions in temper tantrums, 4 (3.0%) had reductions in stereotypical behavior, and 1 (0.8%) exhibited less self-harm. In addition, 4 (3.0%) patients started to sleep better, with resolution of their insomnia. Overall, 83 of the 133 patients (62.4%) reported a CGI-I score of 1 (“very much improved”) and 27 (20.3%) reported a CGI-I score of 2 (“much improved”) at their last visit.

## 4. Discussion

This retrospective cohort study evaluated the safety profile of atomoxetine treatment in 133 children aged 3–6 years with ADHD comorbid with ASD. Approximately one third (37.6%) of the patients reported adverse events, with 17.3% discontinuing further treatment as a result. In the remaining 110 patients (82.7%) who completed at least 6 months of treatment, slow titration of the atomoxetine dose to between 1.2 mg/kg/day and 1.8 mg/kg/day enabled the determination of a target dose that was both tolerable and effective for the majority of the patients, with clinically relevant improvements reported in the patients’ ADHD symptoms. Adverse events and side effects were managed according to their severity. Where these were intolerable, atomoxetine treatment was discontinued. Where these events were milder, they were managed by reducing the dose titration schedule from every 2 weeks to every 4 weeks. Attention is critical in learning and socialization, and the co-occurrence of ADHD increases the risk of negative outcomes in children with ASD [11]. In the present study, all 110 of the patients who completed 6 months of atomoxetine treatment reported an increased attention span; this likely underpinned the other gains observed in some of these patients, such as improvements in receptive and expressive language and the ability to follow complex commands. This is consistent with previous reports in this population, in which atomoxetine was shown to improve anxiety, inattention, self-regulation, and social communication [32,33].

In multiple clinical trials performed by Eli Lilly and Company (Indianapolis, IN, USA), atomoxetine has been shown to be effective in the treatment of the core symptoms of ADHD in children and adolescents of 6 to 18 years of age, improving functional outcomes and quality of life [21,34]. In this demographic, the most common adverse events reported were nausea, vomiting, decreased appetite, abdominal pain, fatigue, and somnolence. Consistent with the results from these clinical trials, gastrointestinal adverse events were the most common safety findings in the present study, although with a higher incidence than that reported in older children [21]; similar discrepancies in the rate of adverse events between older children and children of preschool age have previously been noted for atomoxetine and may be due to differences in pharmacokinetics [35].

In a pooled analysis of these clinical trials (*n* = 2200) by Eli Lilly, an increased risk of suicidal ideation was observed in children and adolescents (0.4% vs. 0% for atomoxetine vs. placebo, with one suicide attempt; all occurred in children aged 12 years or younger during the first month of treatment) [36]. For this reason, the product label includes a warning that pediatric patients need to be monitored carefully for unusual changes in behavior, especially when initiating treatment or when the dose is increased or decreased [21]. A later observational study in the United Kingdom of 4509 children (median age, 11 years) found no evidence of an increased risk of suicidal ideation during atomoxetine treatment compared with the period prior to initiating treatment [37].

To date, three independent studies in the United States have evaluated atomoxetine in patients with ADHD who were younger than 6 years of age [29,30,31]. In the first open-label, 8-week pilot study [29], 22 children aged 5 and 6 years received individualized dosages of atomoxetine that were titrated to a maximum dose of 1.8 mg/kg/day. Statistically and clinically significant improvements were observed in the patients’ ADHD symptoms, and no patients discontinued due to adverse effects or lack of efficacy. The most commonly reported adverse events were mood lability (54.5%) and decreased appetite (50.0%). Limited changes in vital signs were reported, and there were no clinically significant alterations in heart rate, blood pressure, or on ECGs.

A subsequent open-label pilot study built upon the first by including younger patients: 12 patients with ADHD aged 3 to 5 years of age received atomoxetine that was similarly titrated to a maximum dose of 1.8 mg/kg/day, and the children were treated for a mean duration of 8.2 ± 3.0 weeks [30]. As with the first pilot study, atomoxetine treatment resulted in significant improvements in these patients’ ADHD symptoms. The most common adverse effects reported were gastrointestinal in nature (e.g., stomach upset, reduced appetite, and vomiting), and parents also reported high rates of aggression, irritability, and crying. These adverse effects were mostly mild to moderate in severity.

Finally, in an 8-week, double-blind, placebo-controlled study among 101 patients of 5 to 6 years of age, atomoxetine was similarly found to be efficacious and well tolerated [31]. Significant improvements in ADHD symptoms were reported; however, the authors noted that the children exhibited variable responses to treatment, with many remaining significantly impaired by the end of the 8-week study. The most common reported adverse events in the atomoxetine group were gastrointestinal upset (39%) and decreased appetite (30%). Importantly, a systematic review of all data to date in 2015 on atomoxetine treatment in children and adolescents suggested that the response to treatment may build gradually over time, with up to 3 months’ treatment being needed before robust inferences can be drawn [34].

Atomoxetine is mainly metabolized by a highly polymorphic enzyme, Cytochrome P450 2D6, and this may account in large part for the marked heterogeneity observed in the pediatric response to atomoxetine treatment [35,38]. Poor metabolizers face an increased risk of adverse effects, whereas ultra-rapid metabolizers may experience reduced efficacy. For this reason, it is important that the dose be slowly titrated to determine each individual’s target dose that balances these outcomes. Prospective studies evaluating methods of atomoxetine dose optimization (e.g., pharmacogenetic testing and pharmacokinetic modelling) in pediatric patients with a range of metabolizer profiles would be invaluable in determining the most effective and tolerated dosing range [38].

There are several important limitations to consider regarding the present study. Firstly, the short duration of this study prevented any long-term evaluation of atomoxetine safety and effectiveness in this pediatric population. Secondly, the study was a real-world, open-label, retrospective study, which may have introduced potential biases that amplified the perceived safety and effectiveness of atomoxetine in this population (given the real-world, retrospective nature of the study, there was no blinding of clinicians or caregivers, nor was a control arm included).

## 5. Conclusions

Overall, the findings of this study suggest that atomoxetine was well tolerated and may be effective in this large cohort of children aged 3 to 6 years. These results add to the growing body of evidence suggesting the potential role of atomoxetine in treating ADHD associated with ASD in younger children aged 3 to 6 years. However, given the study’s limitations, caution is advised when extrapolating these results to broader populations. It is important to emphasize that atomoxetine administration in children younger than 6 years of age is off-label, and this needs to be clearly communicated to caregivers, along with comprehensive information regarding potential adverse events and side effects. In addition, the incidence of adverse events that may require treatment discontinuation necessitates careful dose titration by clinicians (to a maximum of 1.8 mg/kg/day) and close monitoring by clinicians and patients’ families to ensure patient safety. Continued research is needed to evaluate the generalizability of these findings, particularly in the form of long-term prospective studies on safety, efficacy, and dose optimization.

## Data Availability

The data presented in this study are available on request from the corresponding author. The data are not publicly available due to patient privacy considerations.
